# Prevalence Clinical Syndromes and Outcomes of Cow’s Milk Allergy in Children: A Four-Year Follow-Up

**DOI:** 10.3390/nu17233646

**Published:** 2025-11-21

**Authors:** Ioannis Xinias, Charalampos Agakidis, Theodora Delaporta, Stilianos Xinias, Ioannis Roilidis, Georgios Xinias, Antigoni Mavroudi

**Affiliations:** 1 3rd Department of Pediatrics, Ippokrateion General Hospital, Faculty of Medicine, School of Health Sciences, Aristotle University of Thessaloniki, 54642 Thessaloniki, Greece; xinias@auth.gr (I.X.); dvradelaporta@gmail.com (T.D.); iroilidis@auth.gr (I.R.);; 2 1st Department of Pediatrics, Ippokrateion General Hospital, Faculty of Medicine, School of Health Sciences, Aristotle University of Thessaloniki, 54642 Thessaloniki, Greece

**Keywords:** allergic proctocolitis, breast feeding, cow’s milk allergy, children, FPIES, gastrointestinal, IgE-mediated, infants, tolerance, cow’s milk challenge test

## Abstract

**Background/Objectives:** Cow’s milk allergy (CMA) manifests with various clinical syndromes and has a wide range of symptoms in infants. This study aims to investigate the prevalence, clinical presentation, and outcome of clinical types and subtypes of CMA diagnosed in children within the first 12 months of life. **Methods:** Children with a CMA diagnosis were included in this mixed retrospective and prospective cohort study and were followed up for four years. Data recorded included clinical manifestations, feeding modes, and outcomes. Follow-up included oral cow’s milk (CM) challenge and/or elimination—reintroduction of CM, provided there was parental consent. Also, skin prick test and serum CM-specific IgE were assessed when needed. **Results:** A total of 93 infants (age: 3 days to 24 months) diagnosed with CMA were included. Prevalence was 28% for IgE-mediated CMA and 72%, 49.5%, 18.3%, and 3.7% for non-IgE-mediated CMA and its subtypes, Allergic Proctocolitis (AP), Food Protein induced Enteropathy (FPE), and Food Protein Induced Enterocolitis Syndrome (FPIES), respectively. Main manifestations were gastrointestinal (74%), skin rash (31%), failure to thrive (11.8%), feeding aversion (15.1%), respiratory symptoms (5.4%), and irritability/restlessness (9.7%). Follow-up revealed a high rate of AP and FPE tolerance within the first year, while FPIES and IgE-mediated CMA achieved tolerance at an older age. **Conclusions:** Our study demonstrated the predominance of AP and increased incidence of gastrointestinal involvement. Outcome was good for AP and FPE but less favorable for FPIES and IgE-mediated CMA. Our results, combined with published data, could increase our understanding of CMA-associated syndromes in infants and contribute to the guidance of effective management.

## 1. Introduction

Cow’s milk allergy (CMA) is defined as an immune-mediated response to cow’s milk (CM) proteins that is elicited within a few hours and up to 2 weeks following CM ingestion. It is a significant public health problem, particularly in infancy [1,2,3,4,5]. According to epidemiologic data reported by birth cohort studies, the prevalence of challenge-confirmed CMA during infancy ranged between 1.9% and 4.9%. Specifically, the prevalence of CMA was 1.9%, 2.16%, 2.22%, 2.24%, and 4.9% in Finland, the Isle of Wight, Denmark, the Netherlands, and Norway, respectively [2,3,5]. Some of the most reliable epidemiologic data on CMA in infants is provided by the EuroPrevall multicenter pan-European birth cohort study that was conducted in nine European countries between the years 2005 and 2007 and included a large number of children. The study showed a 0.54% (range 1 to 0.3%) prevalence of positive Oral Cow’s Milk Challenge (OCMC) in infants up to the age of two years [3].

CMA can be divided into three main types: IgE-mediated, non-IgE-mediated, and mixed [2,6]. Patients with IgE-mediated food allergy manifest nausea and vomiting or acute allergic skin reactions, and, in some cases, allergic shock, almost immediately after ingestion of the suspected or proven responsible food. In addition, diarrhea may occur immediately or several hours after exposure to the allergenic food [7]. The non-IgE-mediated allergy is subclassified into three entities/subtypes: Food Protein-Induced Allergic Proctocolitis (FPIAP), referred to in our study as Allergic Proctocolitis (AP), Food Protein-induced Enteropathy (FPE), and Food Protein-Induced Enterocolitis Syndrome (FPIES). The non-IgE-mediated CMA is characterized by a delayed onset of symptoms, some of which overlap with those of functional gastrointestinal (GI) disorders [8].

Suspicion of CMA is mainly clinical, based on detailed history (both family and patient’s) and physical examination, which guide the necessary diagnostic and management strategies and may predict the time of tolerance and other long-term outcomes. However, the diverse and nonspecific symptoms, as well as the lack of specific, reliable biomarkers, are significant obstacles that render clinical diagnosis uncertain [9,10]. SPT and serum CM-specific IgE assessment provide valuable diagnostic support in IgE-mediated CMA and ambiguous cases. Nevertheless, CMA challenge tests with the exclusion of other GI diseases are important steps towards the definitive diagnosis of CMA, especially of the non-IgE-mediated allergic responses [9]. The crucial role of CM challenge tests is underlined by epidemiological studies reporting a discrepancy up to tenfold between challenge test confirmed CMA and symptom-based clinical diagnosis. Specifically, two systematic reviews and metanalyses demonstrated that the prevalence of challenge test identified CMA was about 0.6 to 1% of infants, while symptoms indicating or imitating CMA were present in 6 to 20% of them [2,10]. These figures suggest that CMA in children is over diagnosed when based on clinical data alone. Another issue regarding the difficulties in CMA diagnosis concerns parental reluctance to consent to a challenge test on their infants with IgE-mediated CMA, although the incidence of test-related complications is less than 50% [7]. Moreover, the incidence of severe anaphylactic reactions is very low, while only one case of severe anaphylactic reaction that led to the death of a 9-year-old girl has been reported thus far. This tragic outcome was partly attributed to an unstandardized desensitization approach and could be prevented if appropriate safety measures were implemented [10,11].

The reported outcome of CMA in the pediatric population varies widely, depending mainly on the type or subtype and severity of CMA. In this context, variable rates and the timeframe of tolerance have been reported. For example, FPIES resolution in pediatric populations was attained at a median age of 5.1 years in US subjects with undetectable CM-specific IgE, by 3 years in 90% of an Israeli birth cohort, and by age of 2 years in 100% of a Corean cohort [6,12,13,14,15].

This study aims to describe the prevalence, clinical presentation, and outcome of CMA clinical types and subtypes in children younger than 12 months of age at the time of diagnosis in the current clinical setting.

## 2. Methods

### 2.1. Study Design and Population

This study is a mixed retrospective and prospective study of an infantile cohort diagnosed with CMA between 1 day and 12 months of age, who visited the Pediatric Outpatient Clinic of Allergy or were admitted to the Pediatric Department of Allergy of the Ippokrateion General Hospital of Thessaloniki, Greece, during a two-year period (2019–2021). According to the department protocol, initial assessment included a detailed family history of allergic diseases (e.g., eczema, asthma, and anaphylactic reactions), infant’s medical history, with special focus on allergic and GI disorders, and clinical manifestations of potential CMA, such as mucous and/or bloody stools, diarrhea, vomiting, constipation, respiratory symptoms, and allergic skin rash, as well as anthropometric assessments. Exclusion criteria included suspected or known non-allergic systematic or GI diseases, administration of medications that could potentially obscure challenge test results (e.g., antihistamines and systemic steroids within 7–14 days), missing data or insufficient diagnostic procedures, lost on follow-up, and refusal of parental consent.

### 2.2. Diagnostic Procedures and Definitions

In infants with a history and clinical manifestations indicative of CMA, diagnosis was confirmed by either an open OCMC test or a period of CM avoidance followed by reintroduction. Additional tests included SPT and serum CM-specific IgE, assessed in infants with suspected IgE-mediated CMA, with the exception of infants presented with anaphylaxis, as previous studies suggested [3].

In selected infants with GI symptoms in whom diagnosis remained uncertain despite the appropriate tests, intestinal/bowel biopsies were performed to rule out other GI disorders, after securing parental consent. As soon as the diagnosis was confirmed, CMA was classified as either IgE-mediated CMA or as a subtype of non-IgE-mediated CMA, i.e., AP, FPE, and FPIES, based on clinical and laboratory findings.

Criteria/definitions used for the CMA type and subtype classification were the following [9]:

Diagnosis of IgE-mediated CMA was based on the acute onset of urticaria, angioedema, asthma, and even anaphylaxis in severe cases, following digestion of CM. Positive SPT and specific IgE for CM confirmed the diagnosis. The challenge test was not performed in cases suspected of IgE-CMA due to parental hesitation [5].

Diagnosis of AP was based on the presence of mucus and/or bloody stools in healthy appearing infants starting within the first months of life and exclusion of other causes of bloody stool, such as rectal fissures, intussusception, infectious colitis, and necrotizing enterocolitis [16]. Lack of vomiting or profound diarrhea, with symptom resolution within two weeks after withdrawal of CM, further supported the diagnosis of AP [9].

Diagnosis of FPE was based on a history of chronic diarrhea and vomiting starting from the first year of life, coupled with a challenge test suggestive of CMA. Development of malabsorption and poor weight gain or failure to thrive in infants with persisting symptoms, as well as any concomitant atopic manifestations, further supported the diagnosis of FPE. In selected cases, upper endoscopy with biopsies was performed primarily to rule out celiac disease after obtaining parental consent [9,10,17].

For diagnosis of FPIES, we utilized the criteria developed by the International Consensus Guidelines for diagnosis and management of FPIES that were published by Nowak-Węgrzyn et al. (2017) [18]. In this context, the diagnosis of CMA was based on the presence of one major criterion and at least three minor criteria [18]. In cases where a single episode had occurred, diagnosis was confirmed by an open OCMC [2,18] (see Supplementary Table S1).

### 2.3. Follow-Up

Infants diagnosed with CMA were followed clinically for up to four years to assess the natural history of CMA in each patient. Data collected on follow-up appointments included the type of milk ingestion (breastfeeding or formula), food intake, and the response to milk initiation, with recording of any signs or symptoms indicative of CMA. Tests performed on follow-up included an OCMC test from 6 to 12 months after diagnosis, if parents’ consent. In infants with sustained positive OCMC at 6 to 12 months, the test was repeated between 18 and 24 months. Assessment of serum-specific IgE antibodies and SPTs were also repeated on symptomatic cases when the initial screening test was positive.

### 2.4. Ethical Issues

The study protocol was approved by the Scientific Committee of Ippokrateion General Hospital of Thessaloniki (number of approval 102/23-5-2022) and all procedures were conducted in accordance with the Declaration of Helsinki. Parental written informed consent was obtained before recruitment.

### 2.5. Statistical Analysis

Dichotomous data are presented as counts and percentages of the total study population. Continuous data, such as age at diagnosis and breastfeeding duration, are presented as median and interquartile range (IQR) since their values did not follow a normal distribution (Kolmogorov–Smirnoff test), while the range is also recorded. Data recording and statistical analysis were performed using the Statistical Package for the Social Sciences (SPSS) software for Windows (IBM SPSS Statistics version 23).

## 3. Results

### 3.1. Demographics

The study cohort consisted of a total of 93 children diagnosed with CMA, 51 males (55%) and 42 females (45%). The median age at diagnosis was 1.9 (IQR, 3.4) months, with a range from 3 days to 24 months. A total of 69 (74.2%) infants received mixed feedings (hypoallergenic formula and breastfeeding), 21 (22.6%) were formula-fed only, whereas 3 infants (3.2%) were exclusively breastfed. The median duration of breastfeeding was 3.0 (IQR, 4.0) months (range, 0.5–12 months). Eight children (8.6%) had multiple food allergies. The main characteristics of the study population are summarized in Table 1.

### 3.2. Clinical Manifestations

The GI system was the most affected system (74% of the total study population) (Table 2). The main GI manifestations included bloody and/or mucous stools (52%), persistent diarrhea (24%), vomiting (13%%), anorexia, and feeding aversion (15%), which was severe in 2,6%, suboptimal weight gain or weight loss, and/or stunted linear growth (12%). Additional manifestations included atopic dermatitis (31%), respiratory symptoms (persistent cough and recurring respiratory infections, 5.4%), and irritability or restlessness (9%).

In 69 (74.2%) cases, diagnosis of CMA was confirmed with an OCMC test or elimination and re-introduction of CM. In cases of skin atopic reactions, diagnosis was supported by either SPT, or serum-specific IgE antibody assessment, or both. Endoscopy with intestinal biopsies was performed in six (6.4%) infants with presumed FPE in order to exclude other GI diseases (such as celiac disease or eosinophilic disorders).

### 3.3. Distribution of CMA Types and Subtypes

Of the total cohort, 26 (28%) of cases were classified as IgE-mediated allergy and 67 (72%) as non-IgE-mediated CMA. Regarding the distribution of non-IgE subtypes, 49.5%, 18.3%, and 3.7% of the total population had AP, FPE, and FPIES, respectively. The AP presented mainly with rectal bleeding and mucous stool, the FPE with diarrhea and/or weight loss, and FPIES presented with severe GI symptoms following milk ingestion, mainly nausea, vomiting, and lethargy (Table 3). Manifestations in relation to the CMA-types/subtypes are shown in the Supplementary Table S2.

### 3.4. Follow-Up and Outcome (Table 3)

Follow-up assessment at a mean time of 12 months following diagnosis revealed that in 42 (91%) children with AP, symptoms resolved by the age of 6–12 months, which was confirmed by OCMC in 85% of them. Likewise, 13 (76%) children with FPE were free of symptoms by 6–12 months of age. However, children with FPIES had a more protracted course. Particularly, one (25%) established tolerance at 6–12 months and two more by 18–24 months of age, while one (25%) developed allergic reactions at challenge tests performed at the age of 18–24 months and required continued dietary management. Delayed tolerance was also observed in children with IgE-mediated allergy, of whom 12 (46.2%) continued to have positive SPT at the age of 12–36 months, and 28% exhibited allergic reactions (accidentally), suggesting the need for continued CM avoidance strategies.

## 4. Discussion

Our study provides important information on CMA prevalence and distribution of relevant types and subtypes, as well as the predisposing effect of CM formula feeding, clinical presentation, and outcome of infants and children diagnosed with CMA. In line with previous reports, the median age at CMA diagnosis was 1.9 months (range of 3 days to 12 months) [1,3,19]. Non-IgE-mediated CMA was the predominant type of CMA, with AP being the most common subtype. GI symptoms were the most frequent allergic manifestations, followed by skin reactions. The four-year follow-up confirmed that infants with non-IgE-mediated CMA had a more favorable outcome, with a higher proportion of patients with AP and FPE acquiring tolerance within 12 months.

Several studies reported that IgE-mediated CMA was more prevalent than the non-IgE-mediated type [1,3]. The predominance of IgE-mediated CMA in infants is also supported by the EuroPrevall multicenter pan-European birth cohort study, which demonstrated an overall prevalence of CMA of 0.54% with an IgE-mediated CMA prevalence of 0.44%. In addition, the EuroPrevall study reported that 23.6% of participants with CMA had a non-IgE-mediated CMA, defined by the absence of CM-specific serum IgE titers [2,3]. Varying prevalences were also reported by each of the nine countries participating in the EuroPrevall study. Interestingly, only the United Kingdom, the Netherlands, Poland, and Italy identified children with non-IgE-mediated CMA [3]. Overall, the adjusted incidence of non-IgE CMA was 0.13% and 0.72% in the Italian and United Kingdom birth cohorts, respectively. Moreover, non-IgE-mediated CMA prevailed over IgE-CMA both in the United Kingdom (56.3 vs. 43.7%) and Swedish cohorts (7% vs. 3%) [3,20]. Likewise, we found a predominance of non-IgE over IgE-mediated CMA in our study population (72 vs. 28%) that could be partly attributed to the high incidence of GI symptoms in non-IgE-mediated cases that worried the parents who sought medical advice.

Diagnosis of IgE-mediated CMA was based on clinical symptoms indicative of atopy, such as skin manifestations and asthma, SPT, and serum levels of specific for CM IgE suggestive of IgE-mediated CMA. The OCMC test was not performed because parents refused consent. Of note, acceptance of the OCMC test was totally the parents’ decision following our discussion, where we informed them about all relevant issues, including the risks. Although we underlined that the risk of complications is less than 50%, while severe fatal anaphylactic reactions are extremely rare and can be prevented with appropriate safety measures [7,10], parents of IgE-CMA infants refused to consent. The first death related to CM desensitization was reported by Mondello et al. in 2021 [11] in a 9-year-old girl from southwestern Ontario, Canada, who was allergic to dairy and had asthma. Mondello et al. refer to comments by Dr. Douglas Mack, who co-owns Halton Pediatric Allergy near Toronto, suggesting that this tragic death was the first known fatality in the world related to dairy desensitization. The severe adverse outcome was partially attributed to an unstandardized approach to baked milk intake [11]. It should be noted that previous studies showed that not all patients with negative challenge tests comply with recommendations to widen their intake of dairy products, albeit continue the restrictive diet [10]. For example, Dunlop et al. reported that 28% of patients recommended to receive baked milk continued to avoid any form of CM 2 to 7 years later [21]. Further analysis of the non-IgE CMA cases revealed that the most common subtype in our study population was the AP, accounting for about 50% of the total CMA cases [9,16,17]. Although AP is the most frequent cause of rectal bleeding in infants, its prevalence remains unclear [16,17,18,22]. Available data showed a prevalence range from 0.16% in a large Israeli birth cohort [23] to 17% in a healthy unselected population [22] and up to 64% in infants with rectal bleeding [6,22,23,24,25]. The prevalence of AP found in our study seems to be high. However, diagnostic criteria were compatible with expert suggestions. Specifically, diagnosis was based on the characteristic clinical manifestations, and either the open OCMC test (performed in most cases) or CM elimination from the infant’s diet followed by subsequent re-introduction. In addition, the high prevalence of AP is in line with statements by many authors that this is the most common CMA-associated subtype, but its prevalence has not been fully elucidated yet [26]. Moreover, we cannot compare our results with those reported by previous studies, because they refer to different populations, i.e., to the general pediatric population or birth cohorts. Finally, the great diversity regarding the reported prevalence of CMA subtypes in various countries is very well known. Many previous authors support a high frequency of allergic proctocolitis and the variation between reports from different countries. Specifically, Barni et al. reported: “*the real prevalence and incidence of allergic proctocolitis is not known, although it is among the most frequent causes of rectal bleeding in children. The epidemiology of allergic proctocolitis across studies in the literature varies widely, probably due to differences in case identification, methodology…*” [26]. Additional studies also suggest a high incidence of allergic proctocolitis [16].

FPE mainly affects the small intestine, leading to chronic diarrhea without causing severe dehydration or metabolic derangements. Epidemiology data are almost lacking [17,27]. A survey in a Finnish cohort of children with GI complaints estimated the prevalence of CMA-associated FPE at 2.2% [28]. Our study showed that FPE was the second most common non-IgE CMA subtype, accounting for 18% of the total CMA cases. Diagnosis of FPE is mainly clinical, as there is no single reliable laboratory diagnostic biomarker discovered yet. In this context, intestinal biopsy is an important diagnostic tool. In our study, endoscopy with intestinal biopsies was performed only in a few cases (*n* = 6) due to parental refusal to provide consent. Nevertheless, our study population met every characteristic manifestation proposed by previous authors [27]. Specifically, all patients were less than nine months of age at initial diagnosis, presented with GI symptoms, including failure to thrive and vomiting after repetitive ingestion of CM, and the symptoms resolved following implementation of the CM elimination diet [6,27].

FPIES is a rare non-IgE-mediated CMA subtype, characterized by severe GI symptoms [13,18]. CM is the most common food associated with FPIES, accounting for about 44% of all FPIES cases [13]. Several definitions for FPIES have been proposed by previous authors. In the current study, we utilized the definition reported by the International Consensus Guidelines for the diagnosis and management of FPIES [18]. As expected, the prevalence of CMA-induced FPIES in our infantile population with CMA (3.7%) was higher than the reported cumulated prevalence of FPIES in general pediatric populations or birth cohorts (range 0.015 to 0.7%) [23,29,30,31,32,33].

Formula feeding is an important risk factor of CMA [2,12,14,23,29,31,34,35]. In contrast, breast milk, recognized for its unique beneficial composition, has been advocated as the best feeding choice for the first six months of life in all infants, especially those with CMA [2]. In fact, a review by Nowak-Wegrzyn et al. (2020) reported that FPIES is very rarely manifested in exclusively breast-fed infants, as breast milk appears to offer protection from a full-blown expression of FPIES [14,34]. Another review by Greer et al. (2019) reported the protective effect of breastfeeding for at least 3–4 months against wheezing in the first two years of life and potentially against childhood asthma, although no definitive conclusion could be reached [36]. On the other hand, bottle feeding at maternity hospital was identified as an independent risk factor of CMA (OR = 1.81 [CI 1.27; 2.59] [37].

However, the protective effect of breastfeeding against CMA has been questioned by other authors who reported a CMA prevalence of 0.5 to 2.1% in infants exclusively breastfed for at least 3 months [33,38,39,40]. The development of CMA in exclusively breast-fed infants was attributed to exposure to CM allergenic proteins (albumin, β-lactoglobulin) excreted in breast milk, which may sensitize predisposed neonates [2,38,40,41,42,43]. Nevertheless, this view was placed in question by a recent systematic review that examined the excretion of food proteins in human milk. It was found that levels of all CM proteins in most human milk samples were lower than the eliciting dose and that the probability of triggering an IgE-mediated CM allergic reaction in a breastfed allergic infant is less than 1:1000 [43]. Based on available data, guidelines published by the ESPGHAN GI Committee in 2012 recommend strict avoidance of allergens by the child and probably the breastfeeding mother, in order to prevent eventual adverse effects of a potentially allergenic CM protein excretion in maternal breast milk, although a maternal restrictive diet is not always considered necessary [2,38,43]. In accordance with ESPGHAN guidelines, participants in our study were fed hypoallergenic formulas either as an exclusive feeding mode (22.6%) or as mixed feeding (formula and breastfeeding, 74%). Moreover, breastfeeding mothers followed an elimination diet in cases where persisting CMA manifestations in their infants were evident.

The prevalence of CMA symptoms varies widely among studies. Some studies reported a higher prevalence of skin manifestations ranging from 50 to 90% [3,4,33,44] followed by GI symptoms with a prevalence from 13 to 59% [3,33,44]. In contrast, other studies in CMA-infants showed that the GI system was affected more commonly than the skin, with an overall prevalence rate of 32–65% and 4.5–60% for GI and skin symptoms, respectively [4,5,45]. The prevalence of GI symptoms in our study population, particularly mucous bloody stools (48%), diarrhea (23.9%), and vomiting (12%), was in accordance with those reported by previous studies, particularly regarding non-IgE-mediated allergies (35–50% of CMA cases) [2,4]. However, overall GI involvement in our study (74%) was higher than previously reported [5]. This discrepancy may be attributed to the more worrisome nature of GI symptoms compared to skin reactions, urging parents to seek immediate medical advice. In our study, skin manifestations were the most common non-GI symptoms (31.2%), while respiratory involvement (5%) was rare.

The potential effect of CMA on growth is another concern. Thus far, available data focusing on the association of CMA with growth is controversial. Early studies failed to demonstrate any significant difference in growth between children with IgE-mediated CMA and controls [46,47]. In contrast, many other studies reported increased frequency of weight loss or low weight gain and/or suboptimal linear growth in approximately 16–23% of CMA cases, particularly in younger infants and in the co-existence of eczema [8,45,48,49]. These reports are in keeping with our results showing that 12% of CMA infants were presented with failure to thrive.

Concerning the outcome of CMA in infants and older children, the general consensus is that IgE-mediated allergies and FPIES exhibit more persistent symptoms, whereas AP and FPE tend to resolve earlier [6,13,16,17,25,26,32]. Previous studies demonstrated that about 57% of children with IgE-mediated CMA attained tolerance within one year after diagnosis [3], while ≥90% were tolerant to CM by the age of 6 years [4]. In addition, Skripak et al. reported that IgE-mediated CMA resolved in only 19% and 42% of children and adolescents by 4 and 8 years, respectively [50]. Of our study population with IgE-mediated CMA, 46% attained tolerance by the age of 12–36 months.

Symptoms of AP and FPE resolved in 91 and 76%, respectively, of our study population by the age of 6–12 months, which aligns with current literature. Specifically, Salvatore et al. reported that rectal bleeding usually resolves spontaneously in 20% of CMA-infants and tolerance is acquired within 3 and 12 months, rarely persisting up to 3 years of age [51]. Similar results were obtained by a recent study by Vallianatou et al., who found that 96.5% of 57 infants with AP achieved tolerance at a mean age of 6.3 months [52].

On the other hand, FPIES takes the longest to resolve. We found that FPIES-CMA infants attained tolerance by 6–12 months of age at a rate of 25%, which increased to 75% by 18–24 months of age. These results are in line with the reported delayed tolerance acquisition by US subjects with CMA, of whom 20% and 40% acquired tolerance at the age of 3 and 5.1 years, respectively [13]. However, additional studies in other countries reported higher rates and/or earlier CMA resolution in birth cohorts [12,15,29,53].

### Potential Limitations

Our study design focuses on the prevalence of CMA types and subtypes in a cohort of infants diagnosed with CMA. Therefore, our results can be compared only with data provided by studies enrolling only infants diagnosed with CMA, but not with studies referring to general infantile/pediatric populations or birth cohorts [6,12,13,16]. However, in this context, our study expands our current understanding of CMA types. Another potential limitation is the fact that the DBPCFC, which is considered the gold standard for CMA diagnosis, was not used to confirm CMA diagnosis. This was mainly due to the difficulty of executing DBPCFC tests, which is a widespread concern. [5,7,10,40,54]. In addition, the retrospective nature of the initial evaluation and diagnosis of CMA could potentially skew our results [7,10]. Another potential limitation could be the higher incidence of non-IgE CMA in our study compared to most relevant studies. The increased prevalence of non-IgE CMA in our study population could be partly attributed to the high incidence of GI symptoms in non-IgE-mediated cases that worried the parents who sought medical advice. Nevertheless, certain previous studies also show a higher prevalence of non-IgE CMA than IgE CMA [3,8,20]. The discrepancy between studies could be attributed to variations among studies from different countries concerning several factors including, but not limited to, the CMA type/subtype, symptom severity, age at diagnosis, definitions and diagnostic criteria used, population characteristics, such as family history and feeding mode (formula or breast feeding), maternal medications during pregnancy, maternal diet, environmental, and other factors [19]. The diagnostic difficulties, partly due to the lack of specific biomarkers, maintain the debate regarding the definitive exclusion of a possible overdiagnosis of non-IgE CMA [16]. Another issue complicating CMA diagnosis even further, is the overlap between CMA and common infantile/pediatric GI disease symptoms, especially in the absence of the DBPCMC test. In the context of these drawbacks, the suggestion by CMA experts promoting the implementation of alternative diagnostic procedures, such as the OCMC test or a period of avoidance and subsequent reintroduction of CM [5,7,10,40,54], should be applied for the diagnosis of CMA.

## 5. Conclusions

Our findings are partly consistent with those reported by certain previously published studies that included a similar population to our study participants. However, the considerable differences from other studies regarding the study population render certain comparisons unreliable. Of note, this discrepancy also applies to studies from various countries that reported inconsistent results for CMA-type and -subtype prevalence, frequency of symptoms, as well as rate and time of tolerance acquisition [19]. In the context of difficulties in diagnosis, we cannot definitely exclude a potential overdiagnosis of non-IgE-mediated CMA [16]. Nevertheless, combined analyses of published epidemiology data, including ours, could further expand our knowledge on global variability of CMA prevalence, related types/subtypes, and other characteristics in infants and children. Another issue that should be considered regards the parental reluctance to consent to a challenge test for their infants/children with IgE-mediated CMA. Attending physicians should inform parents that the incidence of challenge test-associated complications is less than 50% [7], severe anaphylactic reactions are very rare, while potentially lethal events are extremely scarce and can be totally prevented with the implementation of appropriate safety measures [10,11]. Overall, reported data underscore the need for personalized management strategies and regular follow-up, particularly in cases with ΙgE-mediated CMA and FPIES. Moreover, the potential impact of sustained CMA on optimal growth and nutrition should not be overlooked.

## Figures and Tables

**Table 1:** Characteristics of the study population.

Characteristic	Number	%
**Male sex** (*n* = 93)	51	54.8
**Feeding mode**		
Mixed *	69	74.2
Formula	21	22.6
Exclusive breastfeeding	3	3.2
**Kind of hypoallergenic formula**		
Extensively hydrolyzed	57	67.1
Fully hydrolyzed	18	21.2
Amino acid formula	10	11.8
**Multiple food allergies**		
Yes	8	8.6
No	13	14.0
Unknown	72	77.4

* mixed feedings, breastfeeding, and formula.

**Table 2:** Main clinical manifestations.

Clinical Manifestations	*n* = 93 *	% *
**Gastrointestinal**	69	74.2
Mucus and/or bloody stool	48	51.6
Diarrhea	22	23.9
Vomiting	12	12.9
Regurgitation	5	5.4
Constipation	3	3.2
Failure to thrive	11	11.8
Anorexia, feeding aversion	14	15.1
**Skin**	29	31.2
**Irritability and/or restlessness**	9	9.7
**Respiratory symptoms**	5	5.4

* Values are expressed as counts and percentages of the total study population.

**Table 3:** CMA type and subtype distribution, age, and rate of tolerance acquisition.

CMA Types/Subtypes	Prevalence		Tolerance Acquisition		
	*n* (93)	% *	Age (Months)	*n*	% ^#^
**IgE-mediated CMA**	26	28.0	12–36	12	46.2
**Overall non-IgE mediated**	67	72.0		56	83.6
Allergic proctocolitis	46	49.5	6–12	42	91.3
Food protein-induced enteropathy	17	18.3	6–12	13	76.5
Food protein-induced enterocolitis syndrome	4	3.7	6–12	1	25.0
			18–24	3	75.0

* % of the total study population; ^#^ % of the respective type or subtype.

## Data Availability

The data presented in this study are available on request from the corresponding author due to privacy.

## Supplementary Materials

The following supporting information can be downloaded at: https://www.mdpi.com/article/10.3390/nu17233646/s1, Supplementary Table S1. Diagnostic criteria of FPIES; Supplementary Table S2. Organs and systems affected by CMA in relation to the type–subtype; CMA dataset2. Row data of the study population.

## Abbreviations

The following abbreviations are used in this manuscript:

AP	Allergic Proctocolitis
CM	Cow’s Milk
CMA	Cow’s Milk Allergy
DBPCFC	Double Blind Placebo Controlled Food Challenge
OCMC	Oral Cow’s Milk Challenge
ESPGHAN	European Society of Pediatric Gastroenterology Hepatology and Nutrition
IgE	Immunoglobulin E
GI	Gastrointestinal
FPE	Food Protein-induced Enteropathy
FPIES	Food Protein-Induced Enterocolitis Syndrome
SPT	Skin Prick Test

## References

[1] Flom J.D., Sicherer S.H. (2019). Epidemiology of Cow’s Milk Allergy. Nutrients.

[2] Vandenplas Y., Broekaert I., Domellöf M., Indrio F., Lapillonne A., Pienar C., Ribes-Koninckx C., Shamir R., Szajewska H., Thapar N. (2024). An ESPGHAN Position Paper on the Diagnosis, Management, and Prevention of Cow’s Milk Allergy. J. Pediatr. Gastroenterol. Nutr..

[3] Schoemaker A.A., Sprikkelman A.B., Grimshaw K.E., Roberts G., Grabenhenrich L., Rosenfeld L., Siegert S., Dubakiene R., Rudzeviciene O., Reche M. (2015). Incidence and Natural History of Challenge-Proven Cow’s Milk Allergy in European Children--EuroPrevall Birth Cohort. Allergy.

[4] Koletzko S., Niggemann B., Arato A., Dias J.A., Heuschkel R., Husby S., Mearin M.L., Papadopoulou A., Ruemmele F.M., Staiano A. (2012). Diagnostic Approach and Management of Cow’s-Milk Protein Allergy in Infants and Children: ESPGHAN GI Committee Practical Guidelines. J. Pediatr. Gastroenterol. Nutr..

[5] Fiocchi A., Brozek J., Schünemann H., Bahna S.L., von Berg A., Beyer K., Bozzola M., Bradsher J., Compalati E., Ebisawa M. (2010). World Allergy Organization (WAO) Diagnosis and Rationale for Action against Cow’s Milk Allergy (DRACMA) Guidelines. Pediatr. Allergy Immunol. Off. Publ. Eur. Soc. Pediatr. Allergy Immunol..

[6] Caubet J.-C., Szajewska H., Shamir R., Nowak-Węgrzyn A. (2017). Non-IgE-Mediated Gastrointestinal Food Allergies in Children. Pediatr. Allergy Immunol. Off. Publ. Eur. Soc. Pediatr. Allergy Immunol..

[7] Santos A.F., Riggioni C., Agache I., Akdis C.A., Akdis M., Alvarez-Perea A., Alvaro-Lozano M., Ballmer-Weber B., Barni S., Beyer K. (2023). EAACI Guidelines on the Diagnosis of IgE-Mediated Food Allergy. Allergy.

[8] Vandenplas Y., Brough H.A., Fiocchi A., Miqdady M., Munasir Z., Salvatore S., Thapar N., Venter C., Vieira M.C., Meyer R. (2021). Current Guidelines and Future Strategies for the Management of Cow’s Milk Allergy. J. Asthma Allergy.

[9] Zubeldia-Varela E., Barker-Tejeda T.C., Blanco-Pérez F., Infante S., Zubeldia J.M., Pérez-Gordo M. (2021). Non-IgE-Mediated Gastrointestinal Food Protein-Induced Allergic Disorders. Clinical Perspectives and Analytical Approaches. Foods.

[10] Hicks A., Fleischer D., Venter C. (2024). The Future of Cow’s Milk Allergy—Milk Ladders in IgE-Mediated Food Allergy. Front. Nutr..

[11] Mondello W. (2021). Girl with Milk Allergy Dies of Severe Reaction Related to Desensitization.

[12] Katz Y., Goldberg M.R., Rajuan N., Cohen A., Leshno M. (2011). The Prevalence and Natural Course of Food Protein-Induced Enterocolitis Syndrome to Cow’s Milk: A Large-Scale, Prospective Population-Based Study. J. Allergy Clin. Immunol..

[13] Caubet J.C., Ford L.S., Sickles L., Järvinen K.M., Sicherer S.H., Sampson H.A., Nowak-Węgrzyn A. (2014). Clinical Features and Resolution of Food Protein-Induced Enterocolitis Syndrome: 10-Year Experience. J. Allergy Clin. Immunol..

[14] Nowak-Wegrzyn A., Berin M.C., Mehr S. (2020). Food Protein-Induced Enterocolitis Syndrome. J. Allergy Clin. Immunol. Pract..

[15] Hwang J.-B., Sohn S.M., Kim A.S. (2009). Prospective Follow-up Oral Food Challenge in Food Protein-Induced Enterocolitis Syndrome. Arch. Dis. Child..

[16] Mennini M., Fiocchi A.G., Cafarotti A., Montesano M., Mauro A., Villa M.P., Di Nardo G. (2020). Food Protein-Induced Allergic Proctocolitis in Infants: Literature Review and Proposal of a Management Protocol. World Allergy Organ. J..

[17] Barni S., Mori F., Pecoraro L., Saretta F., Giovannini M., Arasi S., Liotti L., Mastrorilli C., Klain A., Gelsomino M. (2024). Food Protein-Induced Enteropathy: A Revision for the Clinician. Front. Pediatr..

[18] Nowak-Węgrzyn A., Chehade M., Groetch M.E., Spergel J.M., Wood R.A., Allen K., Atkins D., Bahna S., Barad A.V., Berin C. (2017). International Consensus Guidelines for the Diagnosis and Management of Food Protein–Induced Enterocolitis Syndrome: Executive Summary—Workgroup Report of the Adverse Reactions to Foods Committee, American Academy of Allergy, Asthma & Immunology. J. Allergy Clin. Immunol..

[19] Savage J., Sicherer S., Wood R. (2016). The Natural History of Food Allergy. J. Allergy Clin. Immunol. Pract..

[20] Winberg A., West C.E., Strinnholm Å., Nordström L., Hedman L., Rönmark E. (2015). Assessment of Allergy to Milk, Egg, Cod, and Wheat in Swedish Schoolchildren: A Population Based Cohort Study. PLoS ONE.

[21] Dunlop J.H., Keet C.A., Mudd K., Wood R.A. (2018). Long-Term Follow-Up After Baked Milk Introduction. J. Allergy Clin. Immunol. Pract..

[22] Martin V.M., Virkud Y.V., Seay H., Hickey A., Ndahayo R., Rosow R., Southwick C., Elkort M., Gupta B., Kramer E. (2020). Prospective Assessment of Pediatrician-Diagnosed Food Protein-Induced Allergic Proctocolitis by Gross or Occult Blood. J. Allergy Clin. Immunol. Pract..

[23] Sathya P., Fenton T.R. (2024). Cow’s Milk Protein Allergy in Infants and Children. Paediatr. Child Health.

[24] Xanthakos S.A., Schwimmer J.B., Melin-Aldana H., Rothenberg M.E., Witte D.P., Cohen M.B. (2005). Prevalence and Outcome of Allergic Colitis in Healthy Infants with Rectal Bleeding: A Prospective Cohort Study. J. Pediatr. Gastroenterol. Nutr..

[25] Arvola T., Ruuska T., Keränen J., Hyöty H., Salminen S., Isolauri E. (2006). Rectal Bleeding in Infancy: Clinical, Allergological, and Microbiological Examination. Pediatrics.

[26] Barni S., Mori F., Giovannini M., Liotti L., Mastrorilli C., Pecoraro L., Saretta F., Castagnoli R., Arasi S., Caminiti L. (2023). Allergic Proctocolitis: Literature Review and Proposal of a Diagnostic-Therapeutic Algorithm. Life.

[27] Pundit V.A., Makkoukdji N., Banegas Carballo K.M., Stone F., Satnarine T., Kuhn J., Kleiner G.I., Gans M.D. (2024). A Review of Non-IgE Immune-Mediated Allergic Disorders of the Gastrointestinal Tract. Gastrointest. Disord..

[28] Kokkonen J., Haapalahti M., Tikkanen S., Karttunen R., Savilahti E. (2004). Gastrointestinal Complaints and Diagnosis in Children: A Population-Based Study. Acta Paediatr..

[29] Nowak-Wegrzyn A., Warren C.M., Brown-Whitehorn T., Cianferoni A., Schultz-Matney F., Gupta R.S. (2019). Food Protein-Induced Enterocolitis Syndrome in the US Population-Based Study. J. Allergy Clin. Immunol..

[30] Alonso S.B., Ezquiaga J.G., Berzal P.T., Tardón S.D., San José M.M., López P.A., Bermejo T.B., Teruel S.Q., Echeverría Zudaire L.Á. (2019). Food Protein-Induced Enterocolitis Syndrome: Increased Prevalence of This Great Unknown-Results of the PREVALE Study. J. Allergy Clin. Immunol..

[31] Mehr S., Frith K., Barnes E.H., Campbell D.E., FPIES Study Group (2017). Food Protein-Induced Enterocolitis Syndrome in Australia: A Population-Based Study, 2012–2014. J. Allergy Clin. Immunol..

[32] Katz Y., Goldberg M.R. (2014). Natural History of Food Protein-Induced Enterocolitis Syndrome. Curr. Opin. Allergy Clin. Immunol..

[33] Høst A., Halken S. (1990). A Prospective Study of Cow Milk Allergy in Danish Infants during the First 3 Years of Life. Clinical Course in Relation to Clinical and Immunological Type of Hypersensitivity Reaction. Allergy.

[34] Mathew M., Leeds S., Nowak-Węgrzyn A. (2022). Recent Update in Food Protein-Induced Enterocolitis Syndrome: Pathophysiology, Diagnosis, and Management. Allergy Asthma Immunol. Res..

[35] McBride D., Keil T., Grabenhenrich L., Dubakiene R., Drasutiene G., Fiocchi A., Dahdah L., Sprikkelman A.B., Schoemaker A.A., Roberts G. (2012). The EuroPrevall Birth Cohort Study on Food Allergy: Baseline Characteristics of 12,000 Newborns and Their Families from Nine European Countries. Pediatr. Allergy Immunol. Off. Publ. Eur. Soc. Pediatr. Allergy Immunol..

[36] Greer F.R., Sicherer S.H., Burks A.W., Committee on Nutrition, Section on Allergy and Immunology (2019). The Effects of Early Nutritional Interventions on the Development of Atopic Disease in Infants and Children: The Role of Maternal Dietary Restriction, Breastfeeding, Hydrolyzed Formulas, and Timing of Introduction of Allergenic Complementary Foods. Pediatrics.

[37] Garcette K., Hospital V., Clerson P., Maigret P., Tounian P. (2022). Complementary Bottles during the First Month and Risk of Cow’s Milk Allergy in Breastfed Infants. Acta Paediatr..

[38] Caffarelli C., Giannetti A., Buono E.V., Cunico D., Carbone R., Tonello F., Ricci G. (2025). Cow’s Milk Allergy in Breastfed Infants: What We Need to Know About Mechanisms, Management, and Maternal Role. Nutrients.

[39] Saarinen K.M., Juntunen-Backman K., Järvenpää A.L., Klemetti P., Kuitunen P., Lope L., Renlund M., Siivola M., Vaarala O., Savilahti E. (2000). Breast-Feeding and the Development of Cows’ Milk Protein Allergy. Adv. Exp. Med. Biol..

[40] Meyer R., Chebar Lozinsky A., Fleischer D.M., Vieira M.C., Du Toit G., Vandenplas Y., Dupont C., Knibb R., Uysal P., Cavkaytar O. (2020). Diagnosis and Management of Non-IgE Gastrointestinal Allergies in Breastfed Infants-An EAACI Position Paper. Allergy.

[41] Høst A., Husby S., Osterballe O. (1988). A Prospective Study of Cow’s Milk Allergy in Exclusively Breast-Fed Infants. Incidence, Pathogenetic Role of Early Inadvertent Exposure to Cow’s Milk Formula, and Characterization of Bovine Milk Protein in Human Milk. Acta Paediatr. Scand..

[42] Pier J., Järvinen K.M. (2020). Food Allergy and Breast-Feeding. J. Food Allergy.

[43] Gamirova A., Berbenyuk A., Levina D., Peshko D., Simpson M.R., Azad M.B., Järvinen K.M., Brough H.A., Genuneit J., Greenhawt M. (2022). Food Proteins in Human Breast Milk and Probability of IgE-Mediated Allergic Reaction in Children During Breastfeeding: A Systematic Review. J. Allergy Clin. Immunol. Pract..

[44] Venter C., Brown T., Meyer R., Walsh J., Shah N., Nowak-Węgrzyn A., Chen T.-X., Fleischer D.M., Heine R.G., Levin M. (2017). Better Recognition, Diagnosis and Management of Non-IgE-Mediated Cow’s Milk Allergy in Infancy: iMAP-an International Interpretation of the MAP (Milk Allergy in Primary Care) Guideline. Clin. Transl. Allergy.

[45] Sorensen K., Meyer R., Grimshaw K.E., Cawood A.L., Acosta-Mena D., Stratton R.J. (2022). The Clinical Burden of Cow’s Milk Allergy in Early Childhood: A Retrospective Cohort Study. Immun. Inflamm. Dis..

[46] Agostoni C., Grandi F., Scaglioni S., Giannì M.L., Torcoletti M., Radaelli G., Fiocchi A., Riva E. (2000). Growth Pattern of Breastfed and Nonbreastfed Infants with Atopic Dermatitis in the First Year of Life. Pediatrics.

[47] Seppo L., Korpela R., Lönnerdal B., Metsäniitty L., Juntunen-Backman K., Klemola T., Paganus A., Vanto T. (2005). A Follow-up Study of Nutrient Intake, Nutritional Status, and Growth in Infants with Cow Milk Allergy Fed Either a Soy Formula or an Extensively Hydrolyzed Whey Formula. Am. J. Clin. Nutr..

[48] Flammarion S., Santos C., Guimber D., Jouannic L., Thumerelle C., Gottrand F., Deschildre A. (2011). Diet and Nutritional Status of Children with Food Allergies. Pediatr. Allergy Immunol. Off. Publ. Eur. Soc. Pediatr. Allergy Immunol..

[49] Beck C., Koplin J., Dharmage S., Wake M., Gurrin L., McWilliam V., Tang M., Sun C., Foskey R., Allen K.J. (2016). Persistent Food Allergy and Food Allergy Coexistent with Eczema Is Associated with Reduced Growth in the First 4 Years of Life. J. Allergy Clin. Immunol. Pract..

[50] Skripak J.M., Matsui E.C., Mudd K., Wood R.A. (2007). The Natural History of IgE-Mediated Cow’s Milk Allergy. J. Allergy Clin. Immunol..

[51] Salvatore S., Folegatti A., Ferrigno C., Pensabene L., Agosti M., D’Auria E. (2024). To Diet or Not to Diet This Is the Question in Food-Protein-Induced Allergic Proctocolitis (FPIAP)-A Comprehensive Review of Current Recommendations. Nutrients.

[52] Vallianatou G.N., Douladiris N., Mageiros L., Manousakis E., Zisaki V., Galani M., Xepapadaki P., Taka S., Papadopoulos N.G. (2024). Duration of Food Protein-Induced Allergic Proctocolitis (FPIAP) and the Role of Intestinal Microbiota. Pediatr. Allergy Immunol. Off. Publ. Eur. Soc. Pediatr. Allergy Immunol..

[53] Lemoine A., Colas A.-S., Le S., Delacourt C., Tounian P., Lezmi G. (2022). Food Protein-Induced Enterocolitis Syndrome: A Large French Multicentric Experience. Clin. Transl. Allergy.

[54] NICE Overview, Food Allergy, Quality Standards. https://www.nice.org.uk/guidance/qs118.

