# Supplementary material for: Prevalence Clinical Syndromes and Outcomes of Cow’s Milk Allergy in Children: A Four-Year Follow-Up
Source: Nutrients. 2025 Nov 21;17(23):3646. doi: 10.3390/nu17233646 (PMC12693947; doi:10.3390/nu17233646)
Supplement: Supplementary file 1 [file nutrients-17-03646-s001.zip › nutrients-3950814-supplementary/nutrients-3950814-supplementary.pdf]

Supplementary material

Manuscript title: Prevalence Clinical Syndromes and Outcomes of Cow's Milk Allergy in Children: A Four-Year Follow-up

Diagnostic criteria of FPIES

For diagnosis of FPIES, we utilized the criteria suggested by the International Consensus Guidelines for diagnosis and management of FPIES that were published by Nowak-Węgrzyn et al. (2017) [1]. Diagnosis of CMA was based on the presence of one major criterion and at least three minor of the following criteria shown in Supplementary Table S1 [1,2].

Supplementary Table S1. Diagnostic criteria of FPIES

	Criteria
A. Major criterion	• Repeated vomiting within 1 to 4 hours after exposure to the suspect food and the absence of classic IgE-mediated manifestations including allergic skin manifestations or respiratory symptoms
B. Minor criteria	• At least a second episode of repeated vomiting after ingesting the same suspect food<br>• Repeated vomiting episode 1–4 h after exposure to a different food<br>• Appearance of any of the following four manifestations with any suspected reaction:<br>▪ extreme lethargy<br>▪ marked pallor<br>▪ need for emergency department visit<br>▪ need for intravenous fluid support<br>• Diarrhea within 24 hours (usually 5–10 hours)<br>• Hypotension<br>• Hypothermia

If only a single episode had occurred, diagnosis was confirmed by an open oral cow's milk challenge test [1,2].

References

1. Nowak-Węgrzyn, A.; Chehade, M.; Groetch, M.E.; Spergel, J.M.; Wood, R.A.; Allen, K.; Atkins, D.; Bahna, S.; Barad, A.V.; Berin, C.; et al. International Consensus Guidelines for the Diagnosis and Management of Food Protein–Induced Enterocolitis Syndrome: Executive Summary—Workgroup Report of the Adverse Reactions to Foods Committee, American Academy of Allergy, Asthma & Immunology. *J. Allergy Clin. Immunol.* **2017**, *139*, 1111-1126.e4, doi:10.1016/j.jaci.2016.12.966.
2. Vandenplas, Y.; Broekaert, I.; Domellöf, M.; Indrio, F.; Lapillonne, A.; Pienar, C.; Ribes-Koninckx, C.; Shamir, R.; Szajewska, H.; Thapar, N.; et al. An ESPGHAN Position Paper on the Diagnosis, Management, and Prevention of Cow's Milk Allergy. *J. Pediatr. Gastroenterol. Nutr.* **2024**, *78*, 386–413, doi:10.1097/MPG.0000000000003897.

Supplementary Table S2. Organs and systems affected by CMA in relation to the type-subtype

Manifestations / CMA types-subtypes	Number of patients in CMA types-subtypes	Number with the manifestation	% with the manifestation
**Gastrointestinal**			
IgE-mediated	26	8	30.8
AP	46	44	95.7
FPE	17	14	82.4
FPIES	4	3	75
Overall study patients	93	69	74.2
**Feeding aversion**			
IgE-mediated	26	1	3.8
AP	46	4	8.7
FPE	17	7	41.2
FPIES	4	2	50
Overall study patients	93	14	15.1
**Failure to thrive**			
IgE-mediated	26	4	15.4
AP	46	0	0
FPE	17	5	29.4

FPIES	4	2	50
Overall study patients	93	11	11.8
**Skin**			
IgE-mediated	26	20	76.9
AP	46	7	15.2
FPE	17	2	11.8
FPIES	4	0	0
Overall study patients	93	29	31.2
**Respiratory**			
IgE-mediated	26	3	11.5
AP	46	1	2.2
FPE	17	1	5.9
FPIES	4	0	0
Overall study patients	93	5	5.4
